# Protein expression patterns in primary carcinoma of the vagina

**DOI:** 10.1038/sj.bjc.6601944

**Published:** 2004-06-15

**Authors:** K Hellman, A A Alaiya, K Schedvins, W Steinberg, A-C Hellström, G Auer

**Affiliations:** 1 Department of Gynecologic Oncology, Radiumhemmet, Karolinska Hospital, S-171 76 Stockholm, Sweden; 2 Unit of Cancer Proteomics, Department of Oncology and Pathology, Karolinska Institute and Hospital, Stockholm, Sweden; 3 Division of Obstetrics and Gynecology, Department of Woman and Child Health, Karolinska Institute and Hospital, S-171 76 Stockholm, Sweden; 4 Institution of Cytological Diagnosis (Kloster-Paradiese), Im Stiftsfeld 159494 Soest, Germany

**Keywords:** primary vaginal and cervical cancers, hierarchical cluster analysis, protein expression

## Abstract

Protein patterns in six samples from primary vaginal cancers, in five from normal vaginal tissue and in five primary cervical cancers, were analysed using two-dimensional polyacrylamide gel electrophoresis (2-DE). Protein expression profile was evaluated by computer-assisted image analysis (PDQUEST) and proteins were subsequently identified using matrix-assisted laser desorption/ionisation mass spectrometry. The aim was to analyse the protein expression profiles using the hierarchical clustering method in vaginal carcinoma and to compare them with the protein pattern in cervical carcinoma in order to find a helpful tool for correct classification and for increased biomedical knowledge. Protein expression data of a distinct set of 33 protein spots were differentially expressed. These differences were statistically significant (Mann–Whitney signed-Ranked Test, *P*<0.05) between normal tissue, vaginal and cervical cancer. Furthermore, protein profiles of pairs of primary vaginal and cervical cancers were found to be very similar. Some of the protein spots that have so far been identified include Tropomyosin 1, cytokeratin 5, 15 and 17, Apolipoprotein A1, Annexin V, Glutathione-*S-*transferase. Others are the stress-related proteins, calreticulin, HSP 27 and HSP 70. We conclude that cluster analysis of proteomics data allows accurate discrimination between normal vaginal mucosa, primary vaginal and primary cervical cancer. However, vaginal and cervical carcinomas also appear to be relatively homogeneous in their gene expression, indicating similar carcinogenic pathways. There might, further, be a possibility to identify tumour-specific markers among the proteins that are differentially expressed. The results from this study have to be confirmed by more comprehensive studies in the future.

Primary carcinoma of the vagina (PCV) is a rare disease affecting predominantly postmenopausal women (Pecorelli, 2001). Histologically, the majority of PCV consist of squamous cell carcinomas (Pecorelli, 2001). Owing to the rarity of this disease, little is known about the aetiological and prognostic factors. Like cervical carcinomas, PCV has been shown to be associated with HPV, but only in about 50% of the cases (Daling and Sherman, 1992; Hildesheim *et al* (1997)). The prognosis for PCV is quite poor with an overall 5-year survival rate of about 50%, which is worse than for cervical carcinoma (Pecorelli, 2001). Early detection is crucial for the prognosis.

It has been suggested that vaginal and cervical carcinomas have common aetiology since vaginal tumours often occur as second primary malignancy in patients with a history of cervical dysplasia and/or neoplasia or hysterectomy due to these disorders (Choo and Anderson, 1982; Benedet *et al*, 1983; Brinton *et al*, 1990; Eddy *et al*, 1991; Kirkbride *et al*, 1995). In the clinical situation, it is sometimes difficult to discriminate between cervical and vaginal carcinomas, especially in patients with prior cervical disease. As 95% of the recurrences of cervical carcinoma occur within 5 years, many authors have chosen this limit for the distinction between a recurrent cervical carcinoma and a new primary vaginal carcinoma. Correct diagnosis is of importance for the choice of therapy, prognosis and follow-up. The treatment of choice for primary cervical carcinoma is surgery, sometimes in combination with radiotherapy and chemotherapy, whereas radiotherapy alone is the most common treatment for vaginal carcinoma. The treatment and prognosis of especially vaginal, but also cervical, carcinomas mainly depend upon crude histopathological and clinical findings. There is, thus, a need for additional sensitive markers of prognostic and therapeutic importance and for classification.

Proteomic studies are widely used in the search for new tumour markers

Carcinogenesis is a multistep process leading to the development of multiple cell clones and heterogeneity as a result of tumour cell genetic instability.

Two-dimensional gel electrophoresis (2-DE) has been used to examine heterogeneity in gene expression in tissues from different tumours with a view to find tumour-specific molecular markers. With 2-DE, the complex polypeptide expression is analysed qualitatively as well as quantitatively. Significant differences in the polypeptide expression between tumour tissues and the corresponding normal tissues have been identified, for example in carcinoma of the bladder (Celis *et al*, 2000), breast (Franzen *et al*, 1997), colon-rectum (Stulik *et al*, 2001), lung (Schmid *et al*, 1995) and ovary (Alaiya *et al*, 1999), leading to a possibility to find tumour-specific biological markers.

Cluster analysis, which is a method to describe the similarity between samples based on their pattern of gene expression (Eisen *et al*, 1998), has enabled accurate classification of breast tumour tissues (Dwek and Alaiya, 2003).

The purpose of this study was to characterise the protein expression in PCV and to compare the protein profiles with normal vaginal tissue and primary cervical cancer by using 2-DE in order to point out similarities or differences that might be helpful in the diagnosis/differential diagnosis and that could be indicative for related/unrelated aetiology of these carcinomas.

## MATERIALS AND METHODS

### Patient tissue samples

A total of 16 tissue biopsies (about 3 mm × 3 mm) were analysed consisting of five biopsies from normal vaginal epithelium, six from primary vaginal carcinomas and five from primary cervical carcinomas. For histopathological data, see Table 1

**Table 1:** Clinical and histopathological data

**No**	**Sample**	**Type of sample**	**Histology**	**Grade**	**Stage**	**Age**
1	V24N	Normal vaginal tissue				48
2	V25N	Normal vaginal tissue				70
3	V26N	Normal vaginal tissue				56
4	V37N	Normal vaginal tissue				78
5	V38N	Normal vaginal tissue				78
6	V29T	Vaginal carcinoma	Squamous cell carcinoma		I	83
7	V30T	Vaginal carcinoma	Adenocarcinoma		I	54
8	V31T	Vaginal carcinoma	Squamous cell carcinoma		I	89
9	V32T	Vaginal carcinoma	Squamous cell carcinoma	Low	I	62
10	V33T	Vaginal carcinoma	Squamous cell carcinoma	Medium	I	78
11	V34T	Vaginal carcinoma	Squamous cell carcinoma	High	IIB	75
12	Cx40	Cervical carcinoma	Squamous cell carcinoma	Low	IIB	45
13	Cx41	Cervical carcinoma	Adenocarcinoma		IB2	41
14	Cx42	Cervical carcinoma	Squamous cell carcinoma	Low	IIA	63
15	Cx43	Cervical carcinoma	Squamous cell carcinoma	Medium	IB2	59
16	Cx44	Cervical carcinoma	Squamous cell carcinoma	Medium	IB1	65

. We included the normal vaginal tissue to ensure effective comparison with vaginal cancer. The cervical cancer samples were added in an attempt to elucidate similarities and differences between cervical and vaginal cancers at the proteome level. The 11 tumour biopsies were taken from patients with histopathologically confirmed diagnosis of either vaginal or cervical cancers.

In order to ensure sample representativity, the samples were taken by experienced gynaecologists and gynaecological surgeons. Each tissue sample was macroscopically examined and only representative, non-necrotic tissue samples were used. Furthermore, both cytological and histological evaluations of all the samples were made. Only cases in which both histological and cytological features corresponded with each other were included in the study. We did not focus on the HPV status in this study bearing in mind that the limited number of samples will not permit the drawing of any significant conclusion.

The five normal vaginal biopsies were obtained from the upper part of the vagina approximately 1 cm from the vaginal fornix in postmenopausal women undergoing total hysterectomy for either benign disease or endometrial/ovarian carcinoma. All the fresh tissue samples were snap-frozen in liquid nitrogen until further processing for 2 DE. All samples were obtained with patient consent. One of the vaginal cancer cases (V32T) had been treated with radiation therapy for squamous cell carcinoma of the cervix 35 years ago. None of the other vaginal and cervical cancer cases had a history of prior gynaecological cancer. None of the vaginal cancer cases had a history of vaginal or cervical dysplasia or hysterectomy.

## SAMPLE PREPARATION

All the tissue samples were prepared according to a frozen tissue preparation method (Franzen *et al*, 1991), with slight modification. Briefly, whole tissue biopsies were kept frozen in liquid nitrogen and mechanically homogenised using a pestel and mortal. Each sample was then dissolved in 300–500 *μ*l lysis buffer containing 7 M urea, 2 M thiourea, 4% SDS, reducing agents and protease inhibitors. Protein concentration was determined using the Bradford method (Bradford, 1976).

### Electrophoresis, scanning and image analysis

For each sample, the equivalent of 100 *μ*g total solubilised proteins dissolved in 350 *μ*l volume of rehydration buffer (2% (v v^−1^) IPG-buffer 4-7 linear) was loaded onto a 17 cm IPG-strip 4–7 linear (Bio-Rad, Harcules, CA). This gives better resolution and better overview of protein spots across the entire chosen pH window. In addition, the linear gradient also gives a better estimation of the isoelectric point (pI). Isoelectric focusing was performed for each individual sample to a total of 45.5 kVh using Bio-Rad IEF unit (20°C).

The second dimension was carried out in a 10–13% gradient SDS gel, and proteins were visualised by silver staining (Rabilloud *et al*, 1994). After electrophoresis and staining, only high-quality gels were used. Occasionally, some samples had to be rerun in order to obtain comparable quality with other 2-D gels. Stained gels were scanned at 100 *μ*m resolution using a laser densitometer, and data were analysed using the PDQUEST™ software (version 7.1.0, Bio-Rad). Gel images were compared for qualitative and quantitative differences. Polypeptide quantities were calculated in parts per million (ppm) of the total integrated optical density.

### Mass spectrometry

Protein spots with statistically significant variability in the expression pattern between normal vaginal epithelium, cervical and vaginal cancers were selected for identification by mass spectrometry.

Micropreparative gels for protein identification were prepared essentially like the analytical gels, except that larger amounts (750 *μ*g) of total proteins were loaded and subjected to isoelectric focusing. Following 2-DE, gels were stained using Coomassie colloidal stain. The 2-D gels were analysed by PDQUEST software and spots of interest were manually excised using a clean sharp scalpel and transferred into an eppendorf tube. In-gel digestion for peptide mass fingerprint analysis was carried out manually with trypsin (Oppermann *et al*, 2000), and digests were desalted using Zip Tip (Millipore) as recommended by the manufacturer. Peptides were eluted in 70% acetonitrile/5% formic acid. The eluate was mixed 1 : 1 (v v^−1^) with a saturated matrix solution containing *α*-cyano-4-hydroxycinnamic acid in 30% acetonitrile/0.1% trifluoroacetic acid. Mass mapping of tryptic peptides was performed using MALDI-TOF (above protocol) or Cap-LC-MS/MS on Micromass Q-TOF Ultima mass spectrometer with LC-packings pep Map C18, 75 *μ*m ID column using a gradient of 7–80% (95% acetonitrile and 0.1% formic acid) over a period of 35 min.

Trypsin fragments of masses 842.50 and 2211.10 Da were used as internal standards for spectra calibration. Data generated were screened in databases using a mass tolerance ⩽20 ppm. The licensed ProteinLynx™ Software (Micromass) or mascot was used for mass mapping (http://www.matrixscience.com).

The above protocol of MALDI-TOF analysis has a sensitivity of femtomole amounts of standard 2-DE gel-separated proteins. For a positive identification of the peptide mass fingerprinting, protein scores greater than 72 were considered significant (*P*<0.05), as calculated by the MASCOT scoring algorithm. In addition, at least four matching peptides should be found and more than 50% of the measured masses should match the theoretical peptide fragments.

### Data processing/data analysis

Both quantitative and qualitative 2-DE data sets were generated from PDQUEST, a 2-DE software analysis program.

The data set generated from the matchset based on each individual sample was imported into J-Express as an Excel test format in the form of a data table, with rows representing gels and columns representing spots (Alaiya *et al*, 2000b). The preprocessed data were analysed by hierarchical clustering (Golub *et al*, 1999; Alaiya *et al*, 2002) using the J Express pro software v 2.1 available at http://www.molmine.com.

The J-Express program was primarily designed to analyse microarray data but equally accepts data sets generated from 2-DE analysis.

Hierarchical cluster analysis is a statistical method that is based on measured variables capable of identifying relatively similar groups of samples. This method is based on the strong assumption that an appropriate distance measure for comparing cases has been carefully selected. Thus, the outcome of the clustering analysis depends on the method of calculation of the distance between samples being compared. In this study, the degree of similarity was calculated using the Bray Curtis distance metric and a complete linkage clustering method. The clustering patterns are then represented diagrammatically as dendrograms with trees and branches depicting the degree of sample relatedness. The sets of genes used in the cluster analysis were selected using Student's *t*-test and the Mann–Whitney ranked test analysis (*P*<0.05) between normal vaginal tissue and vaginal cancer samples. A similar analysis was made between groups of primary vaginal cancer and primary cervical cancer. These variables were then used for the classification of the samples into different groups.

Both quantitative and qualitative differences were taken into account for the statistical analysis.

### Correspondence analysis (CA)

We have used correspondence analysis to further evaluate the same data sets used in hierarchical cluster analysis. This was considered as a means to test if the observed set of genes can indeed discriminate the sample groups, bearing in mind the small sample size of this study.

Correspondence analysis is a computational method that is similar to principal component analysis (PCA) with potential to study association between groups of samples based on selected variables.

The data being subjected to CA is presented as two-dimensional graphical display. This method is capable of visualising different structures within a complex data set.

The principle behind the CA is an attempt to group together objects that are similar while dissimilar objects are separated off. The degree of similarity or difference is measured by distances between objects or groups of objects. The analysis has been used to evaluate different complex microarray data (Fellenberg *et al*, 2001).

## RESULTS

### Variation in protein expression between normal vaginal tissue, vaginal cancer and cervical cancer

Tissue samples from 11 cancer patients and five normal vaginal tissues were evaluated. The clinical characteristics of the samples are presented in Table 1. Cells were prepared from fresh-frozen biopsies and extracts were prepared and analysed by 2-DE for both qualitative and quantitative differences in the expression of multiple polypeptides. An average total number of 1373 spots were resolved on 18 × 20 cm 2-D gels and between 75–82% of the spots were matched between all the gels. Gel spots were visualised using silver staining.

Marked quantitative and qualitative changes were observed in the protein expression pattern between normal samples, vaginal cancer and cervical cancer samples. In contrast, differential protein expression data revealed similar expression profiles comparing vaginal and cervical cancer samples compared with normal samples (data not shown). This similarity in protein expression between vaginal and cervical cancers was observed using the correlation analyses between pairs of samples. When pairs of vaginal and cervical samples were compared, an average correlation coefficient of 0.68 was observed, compared with 0.62 and 0.55 for pairs of normal *vs* vaginal cancer and normal *vs* cervical cancers, respectively (Table 2

**Table 2:** Correlation analysis of 2-DE gels from normal, vaginal ancer and cancer of the cervix

**Samples**	**Correlation coefficient (*r*)**
Group correlation among normal tissues	0.70 (*n*=4 pairs)
Group correlation among vaginal cancers	0.76 (*n*=6 pairs)
Group correlation among cervical cancers	0.79 (*n*=6 pairs)
Correlation among pairs of vaginal and cervical cancers	0.68 (*n*=13 pairs)
Correlation among pairs of normal and vaginal cancers	0.62 (*n*=10 pairs)
Correlation among pairs of normal and cervical cancers	0.55 (*n*=10 pairs)

). No significant variation was observed between group correlation among pairs of vaginal cancer and cervical cancer samples with correlation coefficients of 0.76 and 0.79, respectively.

As shown in Table 1, the materials included one vaginal adenocarcinoma and one cervical adenocarcinoma. Pairwise comparison of the adenocarcinoma of the vagina and the adenocarcinoma of the cervix, with the respective squamous cell carcinomas, did not show any significant difference in the correlation coefficient analysis (data not shown).

However, this is in line with the high degree of similarity found between different subtypes of common epithelial ovarian tumours where a relatively large number of samples were analysed (Alaiya *et al*, 1999).

**Figure 1:**
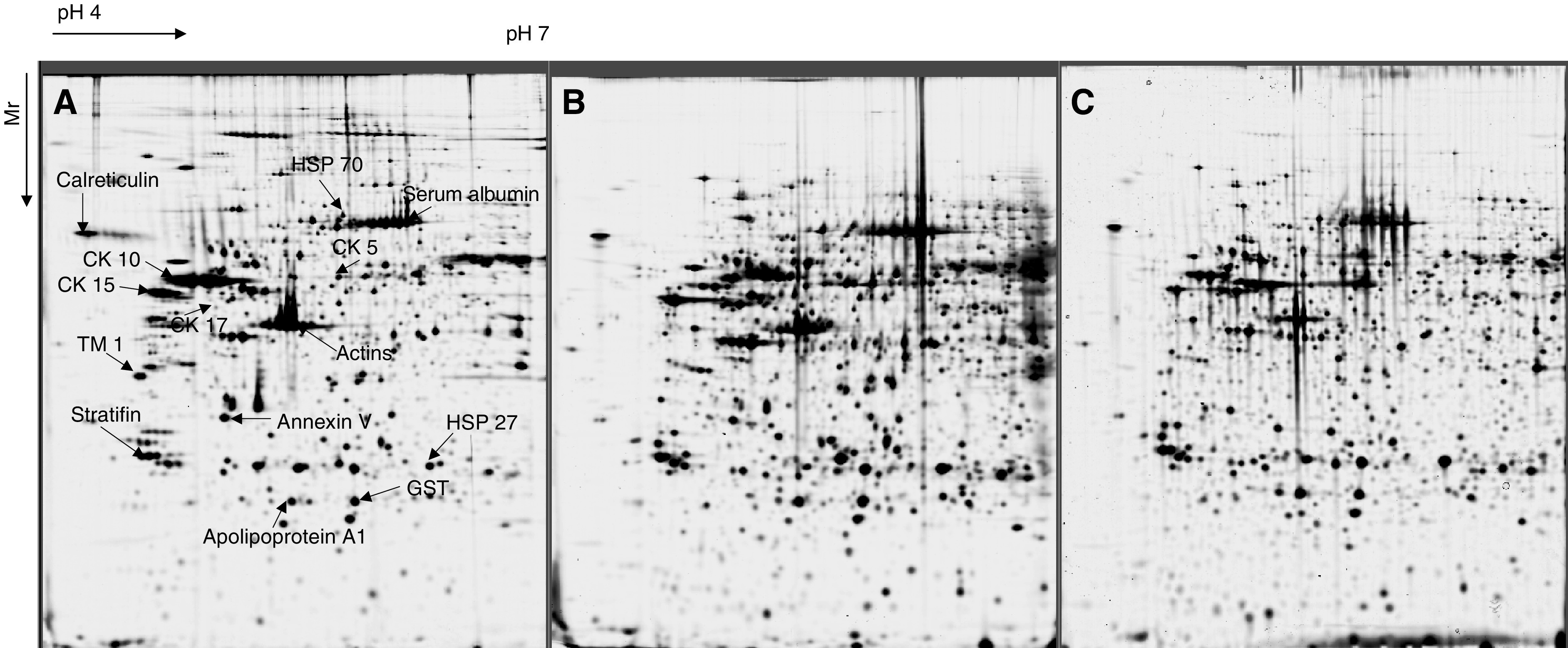
(**A**–**C**) Representative examples of 2-DE gels derived from a normal vagina, primary vaginal cancer and primary cancer of the cervix. Whole-cell lysate was subjected to 2-DE using IPG strips pH 4–7 in the first and 10–13% SDS polyacrylamide gel in the second dimension. Marked are some of the identified proteins: HSP (heat shock proteins), TM (Tropomyosin), CK (Cytokeratin), GST (glutathione-*S-*transferase).

Representative 2-DE maps from normal vaginal tissue, vaginal cancer and cervical cancer are shown in Figure 1

.

### Cluster analysis of differentially expressed proteins in normal vaginal tissue, vaginal cancer and cervical cancer

A total of 67 proteins were differentially expressed in normal vaginal tissue and vaginal/cervical cancers. The differential analysis takes into consideration both qualitative and quantitative changes observed between two sets of samples. This difference was statistically significant using Mann–Whitney analysis (*P*<0.05). A similar analysis was carried out for the three groups of samples using Student's *t*-test analysis, and 94 protein spots differed significantly.

We have used two simple methods of statistical analysis to select the variables that may discriminate the three groups of samples, and proceeded to use these two separate data sets for possible classification of the samples into their respective groups. The samples were correctly classified using the hierarchical cluster analysis (data not shown).

**Figure 2:**
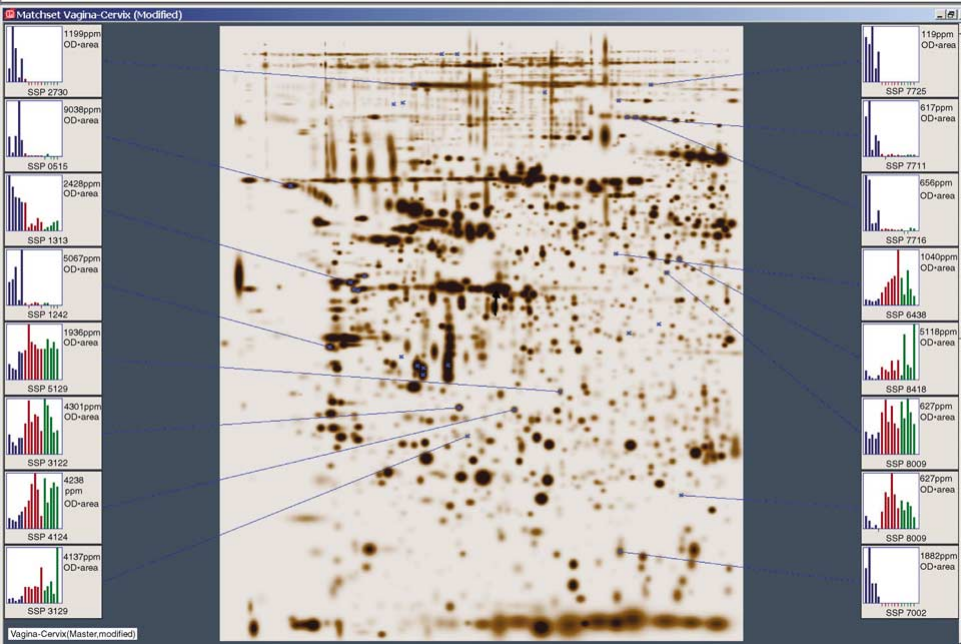
Global analysis of normal vaginal tissue, vaginal cancer and cervical cancer samples using expression data set from 33 polypeptides. Expression levels in all the samples are measured as ppm. Blue=normal, red=vaginal cancer and green=cervical cancer.

In an effort to reduce the data set to a reasonable number, we further examined how many protein spots fall in the intersection of the two data sets, resulting in 33 spots common to both data sets. Of these 33 protein spots, only 11 were upregulated in both cervical and vaginal cancers, whereas the remaining 22 spots were downregulated compared with normal vaginal tissue samples. The differential expressions of some of these protein spots are shown in Figure 2

.

**Figure 3:**
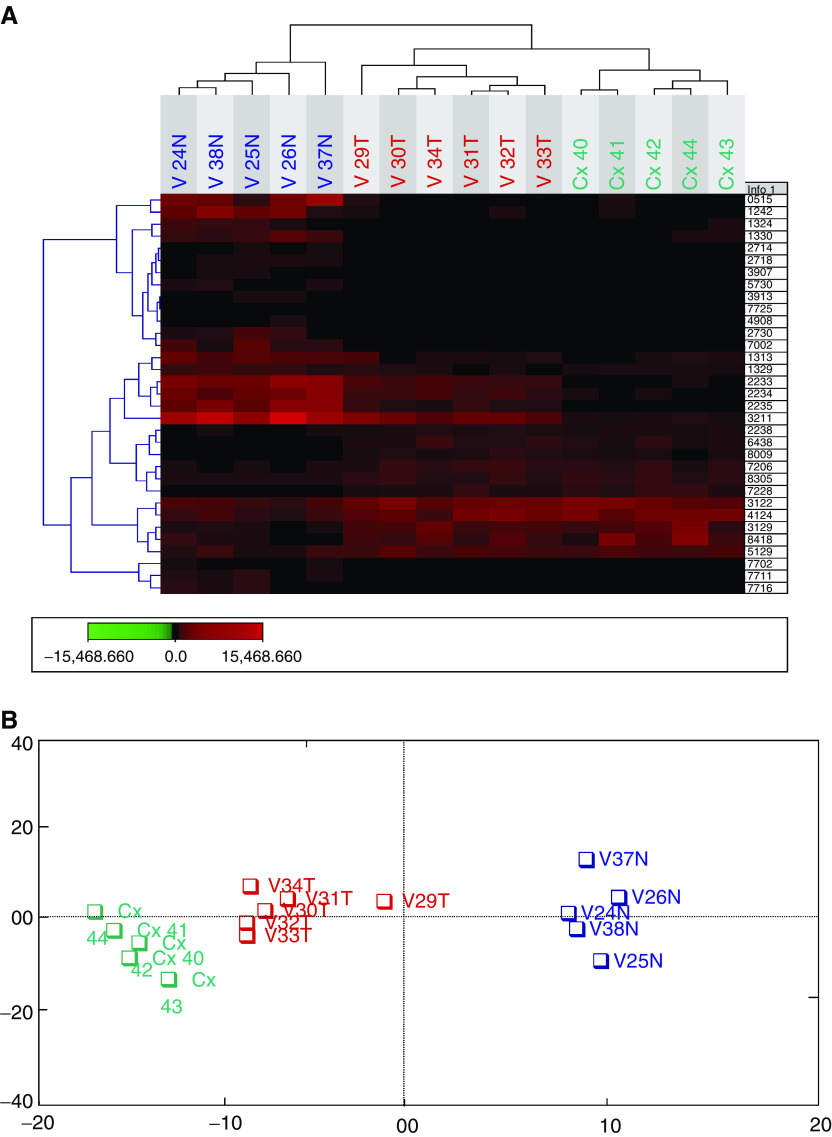
(**A**) Cluster analysis of normal, vaginal cancer and cervical cancer samples using expression data set from 33 polypeptides. (**B**) Correspondence analysis plot of same data set; Blue=normal, red=vaginal cancer and green=cervical cancer.

The 33 spots were used in the cluster analysis of all the samples. As shown in Figure 3a

, all the samples were correctly classified.

Owing to the small sample size of this study, we have used correspondence analysis to evaluate the same data sets used in hierarchical cluster analysis. We have used this as a means to test if the observed set of genes can indeed discriminate the sample groups. As shown in Figure 3b, the samples clustered distinctively, and the relatedness of each sample to each other was presented in a two-dimensional correspondence analysis plot.

This type of analysis allows the identification of potential protein spots that contribute to the overall clustering of the samples.

### Classification of vaginal and cervical cancer

**Figure 4:**
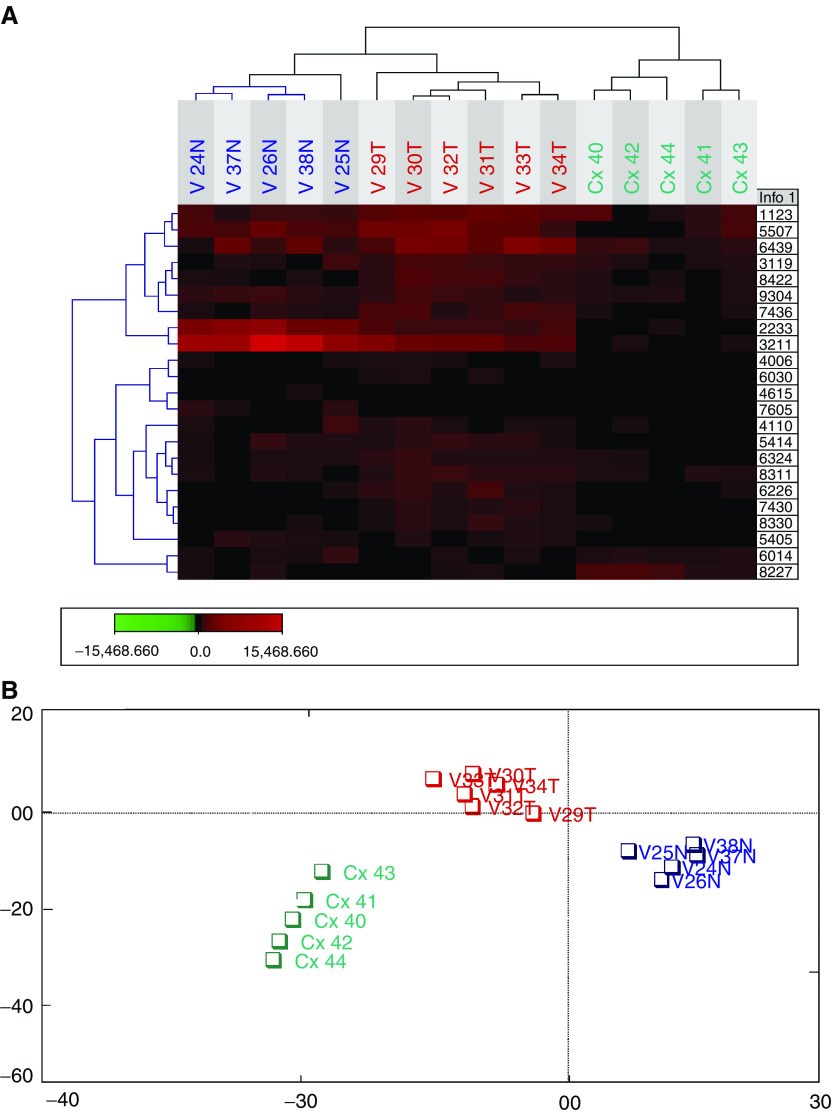
(**A**) Cluster analysis of normal, vaginal cancer and cervical cancer samples using expression data set from 23 polypeptides. (**B**) Correspondence analysis plot of same data set; Blue=normal, red=vaginal cancer and green=cervical cancer.

A total of 23 protein spots were significantly differentially expressed between pairs of 2-DE gels from only vaginal and cervical cancers using both the Mann–Whitney and the *t*-test (*P*<0.05). The expression level of this set of 23 protein spots was used to classify all the samples. Interestingly, all the samples could be correctly classified into three distinct groups (normal tissue, vaginal and cervical cancer), Figure 4

.

### Identification of differentially expressed polypeptides by mass spectrometry

Protein spots with statistically significant variability in the expression pattern between normal vaginal epithelium, cervical and vaginal cancers were selected for identification. Some of these proteins were identified through matching with 2-DE maps of proteins already identified, using bench top MALDI-TOF mass spectrometry. One obvious limitation of working with clinical samples is getting sufficient material for detailed analysis. Therefore, the majority of the protein spots in the data sets for cluster analyses could not be easily identified.

Among the protein spots so far identified are high molecular weight Tropomyosin 1, cytokeratins 5, 15 and 17, Apolipoprotein A1, Annexin V, Glutathione-*S*-transferase. Others are the stress-related proteins, calreticulin, HSP 27 and HSP 70. Some of the identified protein spots are shown in Figure 1.

## DISCUSSION

This is the first proteomic study concerning vaginal carcinoma in the literature. As vaginal carcinoma is a rare disease, the numbers of samples collected in this study are quite few.

In this investigation, we have used hierarchical cluster analysis based on the protein expression in 2-DE to classify vaginal carcinoma. All samples could be correctly classified into three distinct groups (normal tissue, vaginal and cervical cancer). One of the vaginal cancer cases (V32T) had a history of cervical cancer 35 years ago. This case was originally classified as a new primary vaginal carcinoma and not as a recurrent cervical carcinoma due to the long interval between the two carcinomas. In our study, this classification is supported by the results from the cluster analysis, where this vaginal cancer case was classified as a vaginal carcinoma (Figures 3 and 4).

Interestingly, pairs of vaginal cancer and cervical cancer showed to be relatively homogeneous in their protein expression. Studies from ovarian carcinoma have shown large heterogeneity between pairs of different ovarian carcinomas with a correlation coefficient of 0.54 (Alaiya *et al*, 1999). Studies of breast carcinoma have likewise shown large intertumoural heterogeneity, with a correlation coefficient of 0.57 for diploid tumours and 0.48 for aneuploid tumour (Franzen *et al*, 1996). Consequently, pairs of vaginal and cervical carcinomas seem to be more homogeneous than pairs of ovarian and breast carcinomas. This might point at similar genetic alterations and pathways in the carcinogenesis for vaginal and cervical carcinomas. This hypothesis is supported by a recent study by Habermann *et al* (2003) where comparative genomic hybridisation was used to analyse the pattern of genomic imbalances in vaginal squamous cell carcinomas, and revealed that 70% of vaginal carcinomas carry relative increases in copy number that map to chromosome arm 3q. As almost all squamous cell carcinomas of the uterine cervix contain extra copies of chromosome arm 3q (Heselmeyer *et al* (1996)), the pattern of genomic imbalances in PCV is strikingly similar to the one observed in cervical carcinomas.

According to a recent study by Hellman *et al* (2003), there seem to be two types of vaginal carcinoma with age-related aetiology: one type occurring at younger age with aetiological factors similar to cervical carcinoma and another type occurring at older age with different aetiology. This might explain why some vaginal and cervical carcinomas seem to be more homogeneous in their gene expression, whereas others are more heterogeneous.

Previous studies have described marked variations in the expression of cell cycle-related proteins, stress proteins and members of cytoskeletal proteins between benign and malignant epithelial tumours of lung, breast, ovary and prostate gel-separated proteins (Alaiya *et al*, 2000a; Bergman *et al*, 2000). Similarly, in this study, we observed high expression of HSP 27, GST and Apolipoprotein A1 in both cervical and vaginal cancers compared with normal vaginal tissue. In contrast, CK 17, a member of a family of intermediate filament proteins that are characteristic of epithelial cells as well as tropomyosin 1 (TM 1), were upregulated in normal vaginal tissue but not in both vaginal and cervical cancer. Other proteins identified without significantly altered expression levels between the three sample groups are annexin V, actins, calreticulin and Stratifin, a member of the 14-3-3 family proteins.

This observation may indicate that some proteins that are differentially expressed between benign and malignant epithelial tumours may not be similarly altered in some other epithelial tumours such as squamous cell tumours of the vagina and the cervix. The finding of similar expression pattern of some sets of proteins in both squamous cell tumours and other epithelial malignancies may indicate their potential use as markers of malignancy. In this study, we found that 23 spots enabled clustering of almost all of the samples. This set of proteins is evidently interesting for further studies in the search for potential markers, and may give better insight into the aetiology and progression of vaginal and cervical cancers.

According to an earlier study in ovarian carcinoma, cluster analysis of a set of differentially expressed proteins also could be used as a prognostic tool (Alaiya *et al*, 2002). However, this material is too small for survival analysis but might be useful in future studies.

Most methods of statistical analysis are capable of identifying potential marker variables that show significant differential expression between two or more sets of sample groups. However, data sets used in making predictions between two sample groups may potentially be susceptible to data over-fit. This problem is obvious if there are no real biological differences and if the samples being compared are relatively small. It would, therefore, be interesting to test the set of genes used in the learning data to determine whether it can truly differentiate between the two groups when new samples are added. Unfortunately this was not possible to test in the present study because of the small sample size. However, the observed result from the correspondence analysis is in keeping with the cluster analysis data. Despite the limited sample size, the observed result is encouraging and warrants further validation studies.

In conclusion, we have used 2-DE to study protein expression profiles of vaginal and cervical tissue samples and found that hierarchical cluster analysis allowed accurate discrimination between normal vaginal, vaginal and cervical cancer tissue specimens. This study thus indicates that cluster analysis might be utilised for correct classification of the tumours. Further, there might be a possibility to find tumour-specific markers among the differentially expressed proteins.

Vaginal and cervical carcinomas were also found to be quite homogeneous in their protein expression, which might indicate similar aetiological pathways.

## References

[bib1] Alaiya A, Roblick U, Egevad L, Carlsson A, Franzen B, Volz D, Huwendiek S, Linder S, Auer G (2000a) Polypeptide expression in prostate hyperplasia and prostate adenocarcinoma. Anal Cell Pathol 21: 1–9 11254220 10.1155/2000/351963 PMC4618420

[bib2] Alaiya AA, Franzen B, Hagman A, Dysvik B, Roblick UJ, Becker S, Moberger B, Auer G, Linder S (2002) Molecular classification of borderline ovarian tumors using hierarchical cluster analysis of protein expression profiles. Int J Cancer 98: 895–899 11948469 10.1002/ijc.10288

[bib3] Alaiya AA, Franzen B, Hagman A, Silfversward C, Moberger B, Linder S, Auer G (2000b) Classification of human ovarian tumors using multivariate data analysis of polypeptide expression patterns. Int J Cancer 86: 731–736 10797298 10.1002/(sici)1097-0215(20000601)86:5<731::aid-ijc20>3.0.co;2-a

[bib4] Alaiya AA, Franzen B, Moberger B, Silfversward C, Linder S, Auer G (1999) Two-dimensional gel analysis of protein expression in ovarian tumors shows a low degree of intratumoral heterogeneity. Electrophoresis 20: 1039–1046 10344283 10.1002/(SICI)1522-2683(19990101)20:4/5<1039::AID-ELPS1039>3.0.CO;2-4

[bib5] Benedet JL, Murphy KJ, Fairey RN, Boyes DA (1983) Primary invasive carcinoma of the vagina. Obstet Gynecol 62: 715–719 6633996

[bib6] Bergman AC, Benjamin T, Alaiya A, Waltham M, Sakaguchi K, Franzen B, Linder S, Bergman T, Auer G, Appella E, Wirth PJ, Jornvall H (2000) Identification of gel-separated tumor marker proteins by mass spectrometry. Electrophoresis 21: 679–686 10726777 10.1002/(SICI)1522-2683(20000201)21:3<679::AID-ELPS679>3.0.CO;2-A

[bib7] Bradford MM (1976) A rapid and sensitive method for the quantitation of microgram quantities of protein utilizing the principle of protein-dye binding. Anal Biochem 72: 248–254 942051 10.1016/0003-2697(76)90527-3

[bib8] Brinton LA, Nasca PC, Mallin K, Schairer C, Rosenthal J, Rothenberg R, Yordan Jr E, Richart RM (1990) Case–control study of *in situ* and invasive carcinoma of the vagina. Gynecol Oncol 38: 49–54 2354827 10.1016/0090-8258(90)90010-i

[bib9] Celis JE, Wolf H, Ostergaard M (2000) Bladder squamous cell carcinoma biomarkers derived from proteomics. Electrophoresis 21: 2115–2121 10892722 10.1002/1522-2683(20000601)21:11<2115::AID-ELPS2115>3.0.CO;2-K

[bib10] Choo YC, Anderson DG (1982) Neoplasms of the vagina following cervical carcinoma. Gynecol Oncol 14: 125–132 7095583 10.1016/0090-8258(82)90059-2

[bib11] Daling JR, Sherman KJ (1992) Relationship between human papillomavirus infection and tumours of anogenital sites other than the cervix. IARC Sci Publ 119: 223–241 1330912

[bib12] Dwek MV, Alaiya AA (2003) Proteome analysis enables separate clustering of normal breast, benign breast and breast cancer tissues. Br J Cancer 89: 305–307 12865921 10.1038/sj.bjc.6601008 PMC2394238

[bib13] Eddy GL, Marks Jr RD, Miller III MC, Underwood Jr PB (1991) Primary invasive vaginal carcinoma. Am J Obstet Gynecol 165: 292–296; discussion 296–298 1872329 10.1016/0002-9378(91)90081-2

[bib14] Eisen MB, Spellman PT, Brown PO, Botstein D (1998) Cluster analysis and display of genome-wide expression patterns. Proc Natl Acad Sci USA 95: 14863–14868 9843981 10.1073/pnas.95.25.14863 PMC24541

[bib15] Fellenberg K, Hauser N, Brors B, Neutzner A, Hoheisel J, Vingron M (2001) Correspondence analysis applied to micro array data. Proc Natl Acad Sci USA 98: 10781–10786 11535808 10.1073/pnas.181597298 PMC58552

[bib16] Franzen B, Iwabuchi H, Kato H, Lindholm J, Auer G (1991) Two-dimensional polyacrylamide gel electrophoresis of human lung cancer: qualitative aspects of tissue preparation in relation to histopathology. Electrophoresis 12: 509–515 1915243 10.1002/elps.1150120709

[bib17] Franzen B, Linder S, Alaiya AA, Eriksson E, Fujioka K, Bergman AC, Jornvall H, Auer G (1997) Analysis of polypeptide expression in benign and malignant human breast lesions. Electrophoresis 18: 582–587 9150945 10.1002/elps.1150180341

[bib18] Franzen B, Linder S, Alaiya AA, Eriksson E, Uruy K, Hirano T, Okuzawa K, Auer G (1996) Analysis of polypeptide expression in benign and malignant human breast lesions: down-regulation of cytokeratins. Br J Cancer 74: 1632–1638 8932346 10.1038/bjc.1996.600 PMC2074852

[bib19] Golub TR, Slonim DK, Tamayo P, Huard C, Gaasenbeek M, Mesirov JP, Coller H, Loh ML, Downing JR, Caligiuri MA, Bloomfield CD, Lander ES (1999) Molecular classification of cancer: class discovery and class prediction by gene expression monitoring. Science 286: 531–537 10521349 10.1126/science.286.5439.531

[bib20] Habermann JK, Hellman K, Freitag S, Heselmeyer-Haddad K, Hellström A-C, Shah K (2003) A recurrent gain of chromosome arm 3q in primary squamous carcinoma of the vagina. Cancer Genet Cytogenet (accepted) 10.1016/s0165-4608(03)00245-0 14697635

[bib21] Hellman K, Silfversward C, Nilsson B, Hellström A-C, Frankendal B, Pettersson F (2003) Primary carcinoma of the vagina. Factors influencing the age at diagnosis. The Radiumhemmet series 1956–1996. Int J Gynecol Cancer (accepted) 10.1111/j.1048-891x.2004.014310.x 15228423

[bib22] Heselmeyer K, Schrock E, du Manoir S, Blegen H, Shah K, Steinbeck R (1996) Gain of chromosome 3q defines the transition from severe dysplasia to invasive carcinoma of the uterine cervix. Proc Natl Acad Sci USA 93: 479–484 8552665 10.1073/pnas.93.1.479 PMC40262

[bib23] Hildesheim A, Han C-L, Brinton LA, Nasca PC, Richarts RM, Jones RB (1997) Sexually transmitted agents and risk of carcinoma of the vagina. Int J Gynecol Cancer 7: 251–255

[bib24] Kirkbride P, Fyles A, Rawlings GA, Manchul L, Levin W, Murphy KJ, Simm J (1995) Carcinoma of the vagina – experience at the Princess Margaret Hospital (1974–1989). Gynecol Oncol 56: 435–443 7705681 10.1006/gyno.1995.1077

[bib25] Oppermann M, Cols N, Nyman T, Helin J, Saarinen J, Byman I, Toran N, Alaiya AA, Bergman T, Kalkkinen N, Gonzalez-Duarte R, Jornvall H (2000) Identification of foetal brain proteins by two-dimensional gel electrophoresis and mass spectrometry comparison of samples from individuals with or without chromosome 21 trisomy. Eur J Biochem 267: 4713–4719 10903504 10.1046/j.1432-1327.2000.01524.x

[bib26] Pecorelli S (2001) Annual Report on the results of treatment in gynecologic cancer. FIGO 24: 93–95

[bib27] Rabilloud T, Vuillard L, Gilly C, Lawrence JJ (1994) Silver-staining of proteins in polyacrylamide gels: a general overview. Cell Mol Biol (Noisy-le-grand) 40: 57–75 8003936

[bib28] Schmid HR, Schmitter D, Blum P, Miller M, Vonderschmitt D (1995) Lung tumor cells: a multivariate approach to cell classification using two-dimensional protein pattern. Electrophoresis 16: 1961–1968 8586071 10.1002/elps.11501601322

[bib29] Stulik J, Hernychova L, Porkertova S, Knizek J, Macela A, Bures J, Jandik P, Langridge JI, Jungblut PR (2001) Proteome study of colorectal carcinogenesis. Electrophoresis 22: 3019–3025 11565796 10.1002/1522-2683(200108)22:14<3019::AID-ELPS3019>3.0.CO;2-T

